# Loneliness among very old people with and without dementia: prevalence and associated factors in a representative sample

**DOI:** 10.1007/s10433-022-00729-8

**Published:** 2022-09-16

**Authors:** Josefine Lampinen, Mia Conradsson, Fredrica Nyqvist, Birgitta Olofsson, Yngve Gustafson, Ingeborg Nilsson, Håkan Littbrand

**Affiliations:** 1 Department of Community Medicine and Rehabilitation, Occupational Therapy, Umeå University, Umeå, Sweden; 2 Department of Community Medicine and Rehabilitation, Geriatric Medicine, Umeå University, Umeå, Sweden; 3 Faculty of Education and Welfare Studies, Social Policy, Åbo Akademi University, Vaasa, Finland; 4 Department of Nursing, Umeå University, Umeå, Sweden; 5 Department of Surgical and Preoperative Science, Orthopaedics, Umeå University, Umeå, Sweden

**Keywords:** Aged 80 and over, Cross-sectional study, Dementia, Social participation

## Abstract

Loneliness and dementia are common among very old (aged ≥ 80 years) people, but whether the prevalence of loneliness differs between very old people with and without dementia is unknown and few studies have investigated associated factors. The aims of the present study were to compare the prevalence of loneliness between people with and without dementia in a representative sample of very old people, and to investigate factors associated with loneliness in the two groups separately. This population-based study was conducted with data on 1176 people aged 85, 90, and ≥ 95 years (mean age 89.0 ± 4.47 years) from the Umeå 85 + /Gerontological Regional Database study conducted in northern Sweden, during year 2000–2017. Structured interviews and assessments were conducted during home visits. Loneliness was assessed using the question “Do you ever feel lonely?.” Multivariable logistic regression analysis was conducted to identify factors associated with loneliness in participants with and without dementia. The prevalence of loneliness did not differ between people with and without dementia (50.9% and 46.0%, respectively; *p* =  0.13). Seven and 24 of 35 variables were univariately associated with the experience of loneliness in participants with and without dementia, respectively. In the final models, living alone and having depressive symptoms were associated with the experience of loneliness in both study groups. In participants without dementia, living in a nursing home was associated with the experience of less loneliness. These findings contribute with important knowledge when developing strategies to reduce loneliness in this growing age group.

**Supplementary Information:**

The online version contains supplementary material available at 10.1007/s10433-022-00729-8.

## Introduction

The very old (≥ 80 years) age group is growing the fastest worldwide, with its size estimated to increase from 143 to 426 million people between 2019 and 2050 (United Nations 2020). As the prevalence of dementia increases exponentially with age, the number and proportion of people with dementia will also increase significantly in the future. The identification of factors that may facilitate or hinder the ability of very old people (among whom disability and chronic conditions, including dementia, are common) to live well is increasingly important (Clare et al. 2019; Institute of Medicine 2012). Early research suggests that loneliness is more common among very old people than among younger adults (Dykstra 2009), indicating the need to further explore loneliness in very late life.

The feeling of loneliness is subjective, described as unpleasant and distressing, and caused by a discrepancy between an individual’s desired and achieved levels of social relations (Perlman and Peplau 1981). Whereas being alone can be a valued part of everyday life, helping to preserve one’s identity and providing a broader perspective on life (Graneheim and Lundman 2010; Stanley et al. 2017), loneliness is associated with increased risks of reduced engagement in social activities (Dahlberg et al. 2018), physical inactivity (Pels and Kleinert 2016), and morbidities (Cacioppo et al. 2010; Lara et al. 2019; Valtorta et al. 2016) and premature mortality (Holt-Lunstad et al. 2015). Very old people, including those with dementia, are likely more susceptible to the negative effects of loneliness on health and quality of life due to their poorer general health (Donovan and Blazer 2020). Thus, the reduction of loneliness among very old people is a public health issue warranting policy, practice and research attention (Prohaska et al. 2020). Although loneliness and associated social and health risk factors have been studied extensively (Dahlberg et al. 2021), research on loneliness in very old people has been limited (Brittain et al. 2017; Nyqvist et al. 2013), as most studies have focused on older adults in general (Dahlberg et al. 2015, 2021).

As becoming and being very old entail the accumulation of social and health-related changes and losses expected to affect loneliness, consideration of the specific circumstances of very old people in the examination of loneliness is important. For example, only about one-third of 85-year-olds are married or live with someone, and this status is even rarer among ≥ 95-year-olds (Nyqvist et al. 2017). The proportions of very old people with multimorbidity and those living in nursing homes are also greater than are those of younger older people (Bergdahl et al. 2011; Dedering and Henning 2013) as well as the number and proportion of people with dementia (Mathillas et al. 2011). The consequences of dementia, including reduced cognitive and functional ability, fear of failing in social contexts, lack of initiative and interest, socially inappropriate behaviours and neuropsychiatric symptoms such as hallucination, agitation and depression, may interfere with social participation, which includes involvement in activities that provide for interaction with others (Desmarais et al. 2018; Levasseur et al. 2010). Social participation may also be affected negatively by others’ lack of understanding of dementia and how to interact with affected persons (Moyle et al. 2011). Reduced engagement in social activities may increase the risk of loneliness in people with dementia relative to those without this condition. In addition, people with dementia may not remember social visits due to impaired memory, and thus may experience loneliness more often.

Few studies have investigated whether dementia is associated with loneliness (Holmen et al. 2000; Lee et al. 2022). In a study conducted by Holmén et al. (2000) that included people aged ≥ 75 years, the experience of social loneliness (a perceived lack of broader social relationships [Weiss, 1973]) was more common among people with than among those without dementia, but no such difference was observed for emotional loneliness (a perceived lack of intimate relationships [Weiss, 1973]). Lee et al. (2022) obtained similar results, finding that impaired cognitive function (including dementia) was linked to the experience of greater social and emotional loneliness among people aged ≥ 50 years. However, no study has examined whether the prevalence of loneliness differs specifically between very old people with and without dementia. Given the complex consequences of dementia, the social and health-related risk factors for loneliness may differ among people with and without the condition. In the general older adult population, many such risk factors have been identified but few have been associated consistently with loneliness; these factors include not being married/partnered and partner loss, and having a limited social network, a low social activity level, poor self-perceived health and depression (Dahlberg et al. 2021). To our knowledge, only one study has involved multivariable analysis to identify factors associated independently with loneliness among people with dementia (Victor et al. 2020). Similar to the results of a systematic review (Dahlberg et al. 2021), Victor et al. (2020) found that depressive symptoms and living alone were associated with the experience of severe loneliness in this population. They also found that severe loneliness was associated with advanced age (≥ 80 vs. < 65 years), social isolation and reduced quality of life, but not marital status (Victor et al. 2020). Their sample consisted of community-dwelling people with mild to moderate dementia and a mean age of 76 years. Considering the limited amount of research, the focus on younger older people with dementia and the differences in results between Victor et al. (2020) and Dahlberg et al. (2021), further investigation of factors associated with loneliness among very old people, including nursing home residents and those with severe dementia, are needed. In addition, given the variety of factors influencing loneliness (Dahlberg et al. 2021), research on this condition among very old people should be multifactorial. More knowledge about factors associated with loneliness could be important for the development of effective strategies to counteract it in people with and without dementia.

The aims of the present study were to compare the prevalence of loneliness between people with and without dementia in a representative sample of very old people, and to investigate factors associated with loneliness in the two groups separately. We hypothesised that loneliness would be more prevalent among very old people with than among those without dementia, and that associated factors would differ between these groups due to the specific features of problems associated with dementia.

## Methods

### Procedure

This study was conducted with data from the Umeå 85 + /Gerontological Regional Database study, a population-based cohort study conducted to increase knowledge about the health, quality of life, and living conditions of very old people (von Heideken Wagert et al. 2005). Data collection was initiated in the university city of Umeå in 2000 and in five rural municipalities of Västerbotten County (Storuman, Sorsele, Malå, Vilhelmina, and Dorotea), Sweden, in 2002, and repeated every 5 years through 2017. Eligible people were selected from national tax and population registers according to age; every second 85-year-old (from a randomized starting point), every 90-year-old, and every person aged ≥ 95 years received a letter with information about the study and was contacted by a data collector by telephone or in person to obtain informed consent to participation and to plan a home visit. In cases of cognitive impairment, a close relative or otherwise authorized representative was also consulted and provided oral consent, when appropriate. Structured interviews and assessments were conducted with study participants during home visits, which enabled the participation of individuals with severe dementia and/or multimorbidity and those living in nursing homes. Care personnel and/or relatives were also interviewed in cases of cognitive impairment, and all participants’ medical records were reviewed. Data collection was performed by trained assessors with medical knowledge (physicians, nurses, physical therapists, and medical students).

### Participants

**Fig. 1:**
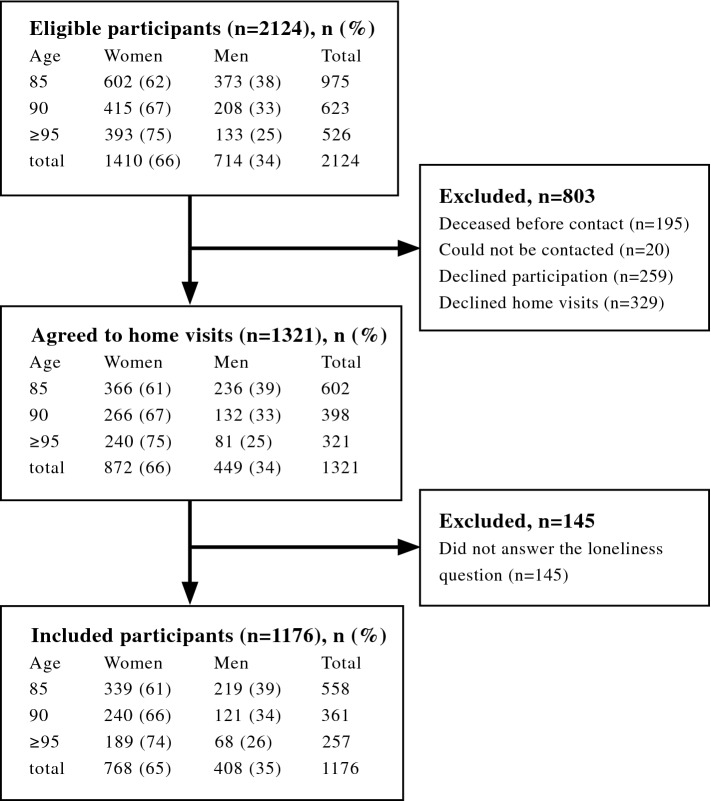
Flow chart of participants through the study

Of 2124 eligible people, 803 were excluded because they died before contact (*n* = 195), could not be contacted (*n* = 20), declined participation (*n* = 259), or declined home visits (*n* = 329). Of 1321 participants who received home visits, 1176 answered a question about loneliness and were included in the present study (Fig. 1). For participants who took part in more than one round of data collection, data from the earliest time point at which they answered the loneliness question were used in the analyses.

### Outcome and study variables

#### Loneliness

The outcome investigated in this study was participants’ loneliness, assessed using the question “Do you ever feel lonely?”. Four response alternatives were provided: “yes, often”; “yes, sometimes”; “no, seldom”; and “no, never.” Following previous research, responses were dichotomized as lonely (“yes, often” and “yes, sometimes”) and not lonely (“no, seldom” and “no, never”) (Nyqvist et al. 2013, 2017).

#### Factors potentially associated with loneliness

Data on participants’ sociodemographic characteristics, social participation, medical conditions, and current medication use were collected during interviews. Baseline variables potentially associated with loneliness, according to previous studies conducted with older people (Dahlberg et al. 2015, 2021; Victor et al. 2020), were chosen. Given the target group, additional variables were added; these included having deceased children, attending a day-care centre, the fear of falling, routine prescriptions, and diagnoses and medical conditions that are common in very old people (Table 1). Widowhood was classified as not widowed, widowed for < 5 years, and widowed for ≥ 5 years. This classification was based on the finding that being widowed for > 5 years protects against loneliness (Brittain et al. 2017). Participants’ living situations were also assessed; those who did not live with a partner or close relative (in the community or in a nursing home) were considered to live alone. The frequency of social contact was measured using two questions: “How many times do you get a visit during a regular week?” and “How many times have you visited others during the previous week?” The numbers of visits received were dichotomized as 0 or 1 and ≥ 2, and the numbers of visits made were dichotomized as 0 and ≥ 1 based on the median split of the data distribution. The perceived frequency of social contacts was measured by two questions: “How often do you experience being visited?” and “How often do you experience visiting others?”. Response options were “often,” “sometimes,” “seldom,” and “never,” and responses were dichotomized as often/sometimes and seldom/never, as in previous research (Nyqvist et al. 2017). Participants’ financial security was assessed with a yes/no question: “Do you consider yourself to have financial security, i.e., you can cope if something unforeseen should happen?” Their educational levels were defined by the number of years of schooling they had received, self-reported or reported by next of kin, and dichotomized as 0–7 and ≥ 8 years. Self-rated health was assessed using the first item of the 36-item Short-Form Health Survey, which is about general health. Responses were dichotomized as good (“excellent,” “very good,” and “good”) and poor (“fair” and “poor”). Cognitive function was assessed using the Mini-Mental State Examination (MMSE), with scores of ≤ 23 of 30 interpreted as indicating impairment (Tombaugh 1992). Participants’ dependence in personal activities of daily living (P-ADL) was assessed using the 10-item Barthel ADL index, with a maximum score of 20 reflecting total independence (Collin et al. 1988). Their dependence in instrumental ADL, including cleaning, shopping, transportation, and cooking, was assessed using the ADL staircase (Sonn & Asberg, 1991). Participants’ depressive symptoms were assessed using the 15-item Geriatric Depression Scale (GDS-15), with scores of ≥ 5 of 15 taken to indicate depression (Conradsson et al. 2013). Their vision and hearing were assessed, and rated as impaired if they were unable to read a word printed in 5-mm capital letters with or without glasses and unable to hear a conversation held in a usual speaking voice from a distance of 1 m with or without a hearing aid, respectively. Participants’ current medication use was assessed using information collected in interviews and from medical records.

**Table 1:** Baseline characteristics and loneliness in participants with and without dementia

	Total (*n* = 1176)	Participants with dementia (*n* = 344)			Participants without dementia (*n* = 832)		
		Lonely 175 (50.9)	Not lonely 169 (49.1)	*p*	Lonely 383 (46.0)	Not lonely 449 (54.0)	*p*
*Sociodemographic and social aspects*							
Age, years	89.0 ± 4.47 (84–103)	90.8 ± 4.97 (84–103)	90.4 ± 4.44 (84–100)	.376	89.0 ± 4.44 (84–103)	87.8 ± 3.90 (84–102)	< .001
*Age group, years*							
85	558 (47.4)	59 (33.7)	52 (30.8)	.137	181 (47.3)	266 (59.2)	< .001
90	361 (30.7)	48 (27.4)	63 (37.3)		120 (31.3)	130 (29.0)	
≥ 95	257 (21.9)	68 (38.9)	54 (32.0)		82 (21.4)	53 (11.8)	
Female gender	768 (65.3)	137 (78.3)	107 (63.3)	.002	267 (69.7)	257 (57.2)	< .001
*Widowhood, n* = *1169*							
Not being widowed	369 (31.6)	31 (17.7)	65 (38.9)	< .001	70 (18.4)	203 (45.5)	< .001
Being widowed ≥ 5 years	673 (57.6)	119 (68.0)	91 (54.5)		249 (65.4)	214 (48.0)	
Being widowed < 5 years	127 (10.9)	25 (14.3)	11 (6.6)		62 (16.3)	29 (6.5)	
Nursing home residents	324 (27.6)	101 (57.7)	89 (52.7)	.346	76 (19.8)	58 (12.9)	.007
Lives alone, *n* = 1162	883 (76.0)	160 (94.7)	124 (73.8)	< .001	348 (91.8)	251 (56.3)	< .001
Having children, *n* = 1175	1039 (88.4)	148 (85.1)	142 (84.0)	.791	343 (89.6)	406 (90.4)	.677
Have deceased children, *n* = 1155	210 (18.2)	33 (19.4)	23 (14.1)	.196	82 (21.7)	72 (16.2)	.045
Born and raised in a place you live today, *n* = 1166	403 (34.6)	60 (34.9)	62 (37.1)	.667	134 (35.1)	147 (33.0)	.536
Education 0–7 years, *n* = 1171	828 (70.7)	134 (78.4)	133 (79.2)	.856	265 (69.2)	296 (65.9)	.316
Having financial security, *n* = 1130	1084 (95.9)	144 (93.5)	143 (96.0)	.337	360 (94.5)	437 (98.0)	.007
*Aspects of Social participation*							
Have visited others during the last week, *n* = 1117 (≥ 1 time/week)	509 (45.6)	37 (22.7)	42 (29.4)	.183	184 (48.8)	246 (56.7)	.025
Being visited during the last week, *n* = 1107 (≥ 2 times/week)	668 (60.3)	78 (49.4)	84 (60.4)	.056	232 (61.7)	274 (63.1)	.675
Experience visiting others, *n* = 1142 Often/sometimes,	507 (44.4)	42 (25.1)	52 (33.8)	.090	158 (41.9)	255 (57.4)	< .001
Experience being visited, *n* = 1145 Often/sometimes	863 (75.4)	114 (68.7)	118 (75.6)	.164	274 (72.1)	357 (80.6)	.004
Having a good friend to talk to, *n* = 1148	846 (73.7)	99 (61.9)	109 (68.1)	.241	284 (74.5)	354 (79.2)	.112
Having a family member to talk to, *n* = 1154	1067 (92.5)	141 (87.0)	148 (91.4)	.210	350 (91.6)	428 (95.5)	.020
Someone who can help, *n* = 1144	1061 (92.7)	145 (89.5)	151 (95.0)	.068	348 (92.1)	417 (93.7)	.358
Someone who cares about you, *n* = 1131	1072 (94.8)	141 (89.2)	138 (89.6)	.915	359 (95.2)	434 (98.2)	.016
Attending a day-care centre, *n* = 1145	329 (28.7)	58 (34.9)	51 (30.5)	.392	108 (28.8)	112 (25.6)	.311
*Diagnosis and medical conditions*							
Depressive disorders, *n* = 1174	393 (33.5)	115 (65.7)	71 (42.3)	< .001	141 (36.9)	66 (14.7)	< .001
Cerebrovascular disease, *n* = 1175	232 (19.7)	44 (25.3)	41 (24.3)	.826	60 (15.7)	87 (19.4)	.162
Heart failure, *n* = 1175	330 (28.1)	56 (32.0)	54 (32.1)	.977	109 (28.5)	111 (24.7)	.223
Previous hip fracture, *n* = 1175	117 (10.0)	25 (14.4)	19 (11.2)	.387	38 (9.9)	35 (7.8)	.280
Vision impairment, *n* = 1172	168 (14.3)	45 (25.7)	39 (23.1)	.612	50 (13.1)	34 (7.6)	.008
Hearing impairment, *n* = 1173	168 (14.3)	49 (26.3)	39 (21.3)	.272	46 (11.6)	47 (10.3)	.435
*Routine prescription medications*							
Antidepressants	192 (16.3)	55 (31.4)	50 (29.6)	.711	56 (14.6)	31 (6.9)	< .001
Analgesics	857 (72.9)	142 (81.1)	136 (80.5)	.875	282 (73.6)	297 (66.1)	.019
Antipsychotics	111 (9.4)	33 (18.9)	21 (12.4)	.101	39 (10.2)	18 (4.0)	< .001
*Assessments*							
Self-rated health as good, (SF-36), *n* = 1141	676 (59.2)	79 (47.6)	97 (62.2)	.008	197 (52.4)	303 (68.3)	< .001
Independent in P-ADL, *n* = 1174	589 (50.2)	29 (43.9)	37 (56.1)	.220	227 (43.4)	296 (56.6)	.043
Independent in I-ADL, *n* = 1171	316 (27.0)	7 (4.0)	9 (5.3)	.567	112 (29.5)	188 (42.0)	< .001
MMSE (0–30), *n* = 1171	22.8 ± 5.61 (0–30)	16.3 ± 4.91 (2–29)	16.2 ± 5.09 (0–26)	.912	25.0 ± 3.28 (9–30)	25.9 ± 2.86 (16–30)	< .001
GDS-15, mean, *n* = 1160	3.62 ± 2.59 (0–15)	5.28 ± 2.80 (1–13)	3.24 ± 2.36 (0–13)	< .001	4.32 ± 2.73 (0–14)	2.56 ± 1.88 (0–15)	< .001
Fear of falling, *n* = 1125	517 (46.0)	91 (57.2)	60 (40.0)	.002	189 (50.4)	177 (40.1)	.003

Data are presented as mean ± standard deviation (range) or *n* (%). Numbers after a characteristic indicate that there are missing assessments.

*SF-36*, 36-item short-form health survey; *P-ADL*, personal activities of daily living (Barthel ADL index); *I-ADL*, instrumental activities of daily living (ADL staircase); *MMSE*, mini-mental state examination, *GDS-15*, 15-item geriatric depression scale. Higher score indicates better status except for GDS-15 where higher scores indicate more depressive symptoms

All medical diagnoses were reviewed by an experienced specialist in geriatric medicine using information from interviews, assessments, prescriptions, and medical records. Dementia and depression disorders were diagnosed according to the Diagnostic and Statistical Manual of Mental Disorders, fourth edition, text revision (American Psychiatric Association 2000). Dementia diagnoses were verified using information from participants’ medical records, prescriptions, and assessments, including the MMSE, GDS-15, Philadelphia Geriatric Center Morale Scale, Life Orientation Scale, Barthel ADL index, ADL staircase, Organic Brain Syndrome Scale, and vision and hearing tests.

#### Statistical analyses

The SPSS software (version 25 for Windows; IBM Corporation, Armonk, NY, USA) was used for all data analyses. The descriptive data are presented as proportions or mean values with standard deviations. Differences in age and sex between study participants and excluded individuals (those who declined participation, declined home visitation or did not answer the loneliness question) were assessed using the independent-samples *t* test and Pearson chi-square test, respectively. Of those participants that were included, baseline differences according to feelings of loneliness (lonely, not lonely) in people with and without dementia, respectively, were tested using independent-samples *t* test and Pearson’s chi-square test. Differences in the proportion of feelings of loneliness in participants with and without dementia was compared using Pearson’s chi-square test.

Multivariable logistic regression analysis was conducted to identify factors associated with the loneliness of participants with and without dementia, respectively. Log-odds linearity was examined graphically for the variables of age, GDS-15, MMSE, and Barthel ADL index scores in the two study groups. As a result of non-linear distribution, age groups were used in the multiple logistic regression models and Barthel ADL index scores were dichotomized as indicating dependence (< 20) and independence (20) in P-ADL. Among measures of depression (diagnoses, antidepressant drug use, and GDS-15 score), only the GDS-15 score was included in the models because it was considered to measure participants’ levels of depressive symptoms in relation to their loneliness. Pearson and Spearman coefficients were used to test for multicollinearity (*r* > 0.60), and revealed strong correlation (*r* = 0.70) between the widowhood and living alone variables among people without dementia. Living alone was selected to be included in the primary multiple regression model as the variable included all those participants living alone, including those living in nursing homes, and not only those living alone based on being widowed. All variables univariately associated with loneliness at the level of *p* < 0.15 (Table 1) were included in the multivariable logistic regression analyses in people with and without dementia, respectively. These variables, organized in blocks, were entered into three models in the following order: (1) sociodemographic characteristics and social aspects; (2) aspects of social participation; and (3) medical conditions, medications used, and assessment results. The results are presented as odds ratios (ORs) with confidence intervals (CIs) and *p* values.

A sensitivity analysis was performed using a stepwise backward deletion strategy to examine data from participants with dementia, due to the high (30%) dropout rate in the third regression model. Stepwise deletion of the variables in the model was performed manually in order of least significance until only significant variables remained. These variables, adjusted for age group and sex, were entered into the final model. All analyses were two-tailed, and *p* < 0.05 was considered to indicate statistical significance.

## Results

Of 1176 individuals included in this study (61.6% of those 1909 persons invited to participate; Fig. 1), 344 (29.3%) had dementia disorders. Individuals who declined participation or home visits (*n* = 588) or did not answer the loneliness question [*n* = 145, 119 (82.1%) of whom had dementia] were more likely to be older (mean age ± SD 89.7 ± 4.83 vs. 89.0 ± 4.47 years, *p* = 0.002) and of female gender (70.3% vs. 65.3%, *p* = 0.025) than were study participants.

Of the participants with dementia, 175 (50.9%) reported experiencing loneliness compared with 383 (46.0%) in participants without dementia (*p* = 0.131). Among participants with dementia, 42 (12.2%) often felt lonely, 133 (38.7%) sometimes felt lonely, 74 (21.5%) seldom felt lonely, and 95 (27.6%) never felt lonely. Among those without dementia, 78 (9.4%) often felt lonely, 305 (36.7%) sometimes felt lonely, 174 (20.9%) seldom felt lonely, and 275 (33.1%) never felt lonely. The prevalences of loneliness among participants with and without dementia were 53.2% and 40.5%, respectively, among 85-year-olds; 43.2% and 48.0%, respectively, among 90-year-olds; and 55.7% and 60.7%, respectively, among those aged ≥ 95 years.

Baseline data are presented in Table 1. In the total sample, 393 (33.5%) participants had depression and 558 (47.4%) experienced loneliness. Three hundred and twenty-four (27.6%) participants lived in nursing homes and of those, 14 lived with their spouses. Relative to those without dementia, participants with dementia were older (mean age 90.6 ± 4.72 vs. 88.4 ± 4.20 years, *p* < 0.001), and larger proportions of these participants were of female gender (70.9% vs. 63.0%, *p* = 0.009), lived alone (84.3% vs. 72.0%, *p* < 0.001), and lived in nursing homes (55.2% vs. 16.1%, *p* < 0.001); they also had a lower mean MMSE score (16.3 ± 5.0 vs. 25.5 ± 3.10, *p* < 0.001) and a higher mean GDS-15 score (4.28 ± 2.76 vs. 3.37 ± 2.47, *p* < 0.001). Seven and 24 of 35 variables were univariately associated with the experience of loneliness in participants with and without dementia, respectively; the two groups shared seven of these variables (female gender, widowhood, living alone, depressive disorders, poor self-rated health, depressive symptoms and fear of falling; all *p* < 0.05; Table 1).

The final multiple logistic regression models for participants with and without dementia included 11 and 21 variables, respectively (Tables 2 and 3). Among participants with dementia (model 3a), the experience of loneliness was associated significantly with living alone (OR, 6.65; 95% CI, 2.26–19.55) and GDS-15 scores reflecting depressive symptoms (OR, 1.41; 95% CI, 1.22–1.62; Table 2). Among those without dementia (model 3b), the experience of loneliness was associated significantly with living in a nursing home (OR, 0.59; 95% CI, 0.35–0.99), living alone (OR, 10.85; 95% CI, 6.51–18.08) and GDS-15 scores reflecting depressive symptoms (OR, 1.41; 95% CI, 1.28–1.56; Table 3).

**Table 2:** Factors associated with loneliness among participants with dementia in multiple logistic regression models

	Model 1a (*n* = 335)			Model 2a (*n* = 268)			Model 3a (*n* = 241)		
	OR	95% CI	*p*	OR	95% CI	*p*	OR	95% CI	*p*
*Sociodemographic and social aspects*									
Age group, years									
85 (reference)									
90	0.56	0.31–0.99	.045	0.64	0.33–1.22	.171	0.58	0.27–1.22	.149
≥ 95	0.72	0.41–1.29	.269	0.75	0.39–1.46	.399	0.85	0.40–1.84	.685
Female gender	1.40	0.80–2.44	.235	1.83	0.96–3.47	.066	1.78	0.86–3.68	.121
Widowhood:									
Not being widowed									
Being widowed ≥ 5 years	1.35	0.73–2.48	.337	0.97	0.46–2.00	.923	0.72	0.31–1.68	.447
Being widowed < 5 years	2.49	1.02–6.09	.045	1.45	0.52–4.06	.481	0.94	0.29–2.98	.909
Lives alone	4.67	1.61–9.47	< .001	4.58	1.77–11.86	.002	6.65	2.26–19.55	< .001
*Aspects of Social participation*									
Being visited during the last week (≥ 2 times/week)				0.65	0.38–1.11	.113	0.84	0.45–1.58	.590
Experience visiting others, Often/sometimes				0.65	0.35–1.18	.154	0.89	0.45–1.79	.752
Someone who can help				0.48	0.17–1.33	.158	0.55	0.17–1.78	.319
*Medications and assessments*									
Neuroleptics							1.49	0.56–3.96	.426
Self-rated health as good, (SF-36)							0.71	0.38–1.33	.288
Depressive symptoms, (GDS-15)							1.41	1.22–1.62	< .001
Fear of falling							1.21	0.65–2.26	.541

*OR*, odds ratio; *CI*, confidence interval; *SF-36*, 36-item short-form health survey; *GDS-15*, 15-item geriatric depression scale. Nagelkerke *R*^2^: model 1a = 0.151, model 2a = 0.180, model 3a = 0.353

**Table 3:** Factors associated with loneliness among participants without dementia in multiple logistic regression models

	Model 1b (*n* = 811)			Model 2b (*n* = 775)			Model 3b (*n* = 747)		
	OR	95% CI	*p*	OR	95% CI	*p*	OR	95% CI	*p*
*Sociodemographic and social aspects*									
Age group, years									
85 (reference)									
90	1.06	0.75–1.51	.741	1.01	0.70–1.47	.942	0.87	0.58–1.32	.512
≥ 95	1.54	0.99–2.40	.054	1.25	0.79–1.99	.340	1.04	0.62–1.76	.876
Female gender	0.86	0.61–1.21	.384	0.83	0.58–1.20	.330	0.85	0.57–1.28	.438
Nursing home residents	0.79	0.52–1.20	.271	0.72	0.46–1.12	.142	0.59	0.35–0.99	.046
Lives alone	9.47	5.99–14.99	< .001	10.12	6.29–16.27	< .001	10.85	6.51–18.08	< .001
Have deceased children	1.14	0.78–1.68	.504	1.12	0.75–1.66	.580	1.01	0.66–1.56	.966
Having financial security	0.37	0.15–0.87	.023	0.41	0.17–0.97	.042	0.49	0.17–1.16	.097
*Aspects of social participation*									
Have visited others during the last week (≥ 1 time/week)				0.98	0.68–1.41	.912	1.20	0.81–1.79	.369
Experience visiting others, Often/sometimes				0.69	0.47–1.01	.056	0.94	0.61–1.44	.762
Experience being visited, Often/sometimes				0.70	0.47–1.05	.083	0.77	0.50–1.20	.253
Having a good friend to talk to				0.84	0.57–1.26	.406	0.91	0.58–1.42	.685
Having a family member to talk to				0.86	0.44–1.69	.669	1.02	0.49–2.10	.963
Someone who cares about you				0.49	0.18–1.34	.165	0.73	0.25–2.14	.563
*Medical conditions, medications and assessments*									
Vision impairment							1.52	0.83–2.80	.175
Analgesics							0.95	0.64–1.39	.777
Antipsychotics							1.65	0.80–3.42	.178
Self-rated health as good, (SF-36)							1.02	0.69–1.51	.930
Independent in P-ADL, (Barthel ADL-index)							1.32	0.86–2.03	.209
Independent in I-ADL, (ADL-staircase)							0.89	0.58–1.38	.607
MMSE (0–30)							0.94	0.89–1.01	.071
Depressive symptoms, (GDS-15)							1.41	1.28–1.56	< .001
Fear of falling							0.97	0.67–1.40	.866

*OR*, odds ratio; *CI*, confidence interval; *SF-36*, 36-item short-form health survey; *P-ADL*, personal activities of daily living (Barthel ADL Index); *I-ADL*, instrumental activities of daily living (ADL staircase); *MMSE*, mini-mental state examination; *GDS-15*, 15-item geriatric depression scale. Nagelkerke *R*^2^: model 1b = 0.235, model 2b = 0.263, model 3b = 0.37

In an additional analysis of data from participants without dementia in which the living alone variable was replaced by the widowhood variable in model 3b, widowhood was associated significantly with the experience of loneliness [OR (95% CI) vs. not widowed: widowed ≥ 5 years, 3.94 (2.53–6.15); widowed < 5 years, 9.45 (5.10–17.51)]. The sensitivity analysis of data from participants with dementia, adjusted for age group and sex, showed the same two variables as in model 3a that were associated significantly with the experience of loneliness (see Supplementary Table 1 in Online Resource 1).

Of the participants with dementia experiencing and not experiencing loneliness, 66 (41.0%) and 28 (18.0%), respectively, desired more friends (*p* < 0.001) and 103 (64.4%) and 71 (45.5%), respectively, wished to meet with family more often (*p* = 0.001). Of the participants without dementia who did and did not experience loneliness, 167 (44.4%) and 109 (24.5%), respectively, desired more friends (*p* < 0.001) and 244 (65.6%) and 200 (45.1%), respectively, wished to meet with family more often (*p* < 0.001).

## Discussion

In this sample of very old people, 47.4% of respondents reported experiencing loneliness sometimes or often. Loneliness was comparably common among respondents with and without dementia, and was associated with living alone and having depressive symptoms in both study groups. In participants without dementia, living in a nursing home was associated with the experience of less loneliness. In addition, when replacing living alone with widowhood in the final model among participants without dementia, being widowed was associated with loneliness. The results indicated that loneliness was associated more strongly with more recent (within 5 years) widowhood.

The finding that the prevalence of loneliness did not differ according to the presence of dementia, although well-known risk factors for loneliness, such as depression and living alone, were more common among participants with dementia, stands in contrast with previous findings from samples including younger older people (Holmén et al. 2000; Lee et al. 2022). Relative to these samples, larger proportions of our participants lived in nursing homes and had severe cognitive impairment. A possible explanation for the lack of a greater loneliness prevalence among participants with dementia may be that people with dementia with more severe cognitive impairment have difficulty assessing temporal aspects of life, and thus may experience their current life situations more as in their earlier lives, and not experience being alone. In support of this interpretation, experiencing loneliness was associated with impaired cognitive function among participants without, but not among those with dementia.

Despite the difference between participants with and without dementia in factors associated with loneliness in univariate analyses, the two groups shared two of the three factors associated independently with loneliness (living alone and having depressive symptoms). These results are in line with previous findings for older people, including very old people (Brittain et al. 2017; Nyqvist et al. 2013; Routasalo et al. 2006) and those with dementia (Victor et al. 2020). The cross-sectional design of the present study prevented us from determining the directionality of these associations, but longitudinal studies have revealed the bidirectionality of the association with depression; i.e., depression increases the risk of loneliness (Brittain et al. 2017; Dahlberg et al. 2015; Donovan and Blazer 2020) and vice versa (Donovan and Blazer 2020). Pritchard et al. (2015) reported that untreated depression in older people may lead to reduced participation in daily activities and, in turn, social isolation, which may lead to feelings of loneliness. To reduce loneliness in society, an understanding of the association between depression and loneliness, screening for depression, and increased awareness of living alone as an established aspect associated with loneliness are important. Furthermore, in contrast to living alone and having depressive symptoms, living in a nursing home was associated with the lack of loneliness in participants without dementia. These results reflect differences in nursing homes’ provision of opportunities for social interaction for people with and without dementia and are in line with findings that such interaction is rare for nursing home residents with dementia (Kolanowski and Litaker 2006; Perrin 1997). This situation may reflect the failure of care staff to make provisions for the occupational needs of people with dementia in an appropriately therapeutic milieu (Perrin 1997). The present results differ from those of Brittain et al. (2017), who reported that living in a nursing home was associated independently with an increased risk of experiencing loneliness in very old people. They adjusted for fewer covariates than we did, which might be one explanation for the difference in results. However, this difference may also reflect differences in nursing home organisation between Sweden and the United Kingdom (Ariaans et al. 2021). In our analysis in which living alone was replaced with widowhood, being recently widowed was associated with a greater risk of loneliness. In agreement with these findings, Brittain et al (2017) found an association between recent widowhood with an increased risk of loneliness in the cross-sectional analysis among very old people, but not in the longitudinal analysis. In addition, in contrast to our study, a protective effect of > 5 years’ widowhood was found.

Most variables associated with loneliness in the two study groups in univariate analyses showed no such association in our multivariable analysis, suggesting that many factors, such as advanced age and female gender or poor self-rated health and ADL dependency, covary. Thus, the measurement and consideration of a wide range of social and health-related factors are important to control for potential confounders in research conducted this field.

In the general older adult population, a limited social network, low social activity level and poor self-perceived health have been consistently associated with an increased risk of experiencing loneliness (Dahlberg et al. 2021). In the present study, poor self-perceived health showed only a univariate association with loneliness among very old people. Data on participants’ social networks and social activity levels were not specifically collected, but the aspects of social participation that were measured, such as visiting in the past week and having a good friend to talk to, were not associated independently with loneliness. Victor et al. (2020) found that advanced age and social isolation (i.e. lack of perceived social support from family and friends), in addition to depression and living alone, were associated independently with loneliness among people with dementia. In the present study, age and factors related to social isolation were not associated independently with loneliness. This difference may be explained by differences in age and morbidity between the study populations. Advanced age and the accumulation of social and health-related changes and losses in our participants may have contributed to many factors covarying in this population.

Our finding of associations between loneliness and the wish to meet with family more often and have more friends adheres to the definition of loneliness as a discrepancy between an individual's desired and achieved levels of social relations (Perlman and Peplau 1981). These results are in line with previous findings regarding the importance of close friends and family relationships to counteract loneliness in younger older people (de Jong Gierveld et al. 2015; Savikko et al. 2005; Taube et al. 2013), and the importance of the quality, rather than quantity, of social relationships (Pinquart and Sorensen 2001). The desire for more friends may reflect participants’ loss of relationships in advanced age due to the deaths of friends who are not easily replaced. In addition, interaction problems related to dementia, such as expressive dysphasia and word-finding difficulties, may be distressing in social contexts and result in the social withdrawal of people with this disease (Farrell et al. 2014). In a qualitative interview-based study conducted with people with dementia and their family-member caregivers, the loneliness of people with dementia was found to be intensified by others’ lack of understanding of dementia and how to interact with them (Moyle et al. 2011). The authors also emphasized the importance of familiar human relationships for the reduction of feelings of loneliness (Moyle et al. 2011). In addition, friends and family members may withdraw from people with dementia due to the difficulty of seeing the disease’s symptoms in a loved one or because they hold stigmatized views of people with dementia (Katsuno 2005). Such withdrawal may have consequences for loneliness that are beyond the control of a person with dementia. For society, important tasks could be to provide family members and friends with information about dementia, and to create arenas for older people aiming to facilitate social interaction where new friendships can be made and impaired cognition is taken into consideration (e.g., activity centres with educated facilitators) (Söderhamn and Landmark 2014).

### Methodological considerations

Data on the prevalence of loneliness were collected during the years 2000–2017, i.e. before the COVID-19 pandemic. The prevalence of loneliness may have increased during the pandemic because of social restrictions. A strength of this study is the sample’s representativeness of very old people, as home visits were conducted to enable the participation of individuals with severe dementia and/or multimorbidity and those who lived in nursing homes. This target group have had limited representation in prior loneliness studies. The results are thus generalizable to individuals aged 85, 90, and ≥ 95 years living in the study area and similar areas in the Nordic region, but they may not be applicable to other very old general populations. Another strength of this study is the extensive data collection, which enabled adjustment for many potential confounders. However, the external validity of the findings may be limited, as individuals who declined participation or home visits and those who did not answer the loneliness question were older, and more of them were of female gender than were study participants. In addition, most individuals who agreed to home visits but did not answer the loneliness question had dementia disorders. Furthermore, we rated loneliness using a single self-reported question (Victor and Bond 2009) instead of an instrument such as the Jong Gierveld Scale (de Jong Gierveld and Kamphuis 1985) or the UCLA Loneliness Scale (Russell 1996). The use of each of these approaches has specific advantages and disadvantages (Victor et al. 2006). The question employed in this study has been used widely and proven to be easy for older people, including those with cognitive impairment, to understand and answer (Tilvis et al. 2012). The question is time-related and measures how often an older person feels lonely (Yang and Victor 2011). Its validity for people with severe cognitive impairments has been questioned (Holmen et al. 2000), but the literature supports the ability of a large proportion of people with such impairments to validly answer questions about their quality of life (Beer et al. 2010; Mozley et al. 1999).

## Conclusion

In very old people, loneliness seems equally prevalent among those with and without dementia, although well-known risk factors for loneliness, such as depression and living alone, were more common among people with dementia. Despite differences between participants with and without dementia in factors associated univariately with loneliness, the two groups shared two of the three factors associated independently with loneliness (living alone and having depressive symptoms). Living in a nursing home was associated with the experience of less loneliness in those without dementia. These findings contribute to important knowledge when developing strategies to reduce loneliness in this growing age group characterized by high risks of loneliness and dementia.

## Supplementary Information

Below is the link to the electronic supplementary material.

Supplementary file1 (PDF 30 KB)

## Data Availability

Data, analytic methods, and study materials for the current study are available from the corresponding author on request.
