# Supplementary material for: Loneliness among very old people with and without dementia: prevalence and associated factors in a representative sample
Source: Eur J Ageing. 2022 Sep 16;19(4):1441–53. doi: 10.1007/s10433-022-00729-8 (PMC9483318; doi:10.1007/s10433-022-00729-8)
Supplement: Supplementary file 1 — Supplementary file1 (PDF 30 KB) [file 10433_2022_729_MOESM1_ESM.pdf]

Loneliness among very old people with and without dementia: prevalence and associated factors in a representative sample

European Journal of Ageing

Josefine Lampinen, Mia Conradsson, Fredrica Nyqvist, Birgitta Olofsson, Yngve Gustafson, Ingeborg Nilsson, and Håkan Littbrand

Josefine Lampinen, Department of Community Medicine and Rehabilitation, Geriatric Medicine, Umeå University, SE-90187 Umeå, Sweden

E-mail: Josefine.lampinen@umu.se

Supplementary Table 1 Additional analysis investigating factors associated with loneliness in participants with dementia in a multiple logistic regression model using a stepwise backward deletion strategy, adjusted for age group and sex. (*n* = 325)

	OR	95% CI	*p*
Age group (years)			
85 (Reference)			
90	0.51	0.27-0.96	.036
≥95	0.64	0.35-1.19	.152
Female gender	1.78	0.99-3.17	.053
Lives alone	5.69	2.42-13.27	<.001
GDS-15	1.40	1.26-1.56	<.001

Note. OR, odds ratio; CI, confidence interval; GDS-15, 15-item Geriatric Depression Scale. Nagelkerke R^2 = 0.295.
